# Comparing Gait with Multiple Physical Asymmetries Using Consolidated Metrics

**DOI:** 10.3389/fnbot.2018.00002

**Published:** 2018-02-13

**Authors:** Tyagi Ramakrishnan, Christina-Anne Lahiff, Kyle B. Reed

**Affiliations:** Rehabilitation Engineering and Electromechanical Design Laboratory, Department of Mechanical Engineering, University of South Florida, Tampa, FL, United States

**Keywords:** gait asymmetry, leg length discrepancy, distal mass, knee orthosis, prosthetic gait

## Abstract

Physical changes such as leg length discrepancy, the addition of a mass at the distal end of the leg, the use of a prosthetic, and stroke frequently result in an asymmetric gait. This paper presents a metric that can potentially serve as a benchmark to categorize and differentiate between multiple asymmetric bipedal gaits. The combined gait asymmetry metric (CGAM) is based on modified Mahalanobis distances, and it utilizes the asymmetries of gait parameters obtained from motion capture and force data recorded during human walking. The gait parameters that were used in this analysis represent spatio-temporal, kinematic, and kinetic parameters. This form of a consolidated metric will help researchers identify overall gait asymmetry by showing them if the overall gait symmetry is improving and avoid the case where one parameter's symmetry is improving while another is getting worse. The CGAM metric successfully served as a measure for overall symmetry with eleven different gait parameters and successfully showed differences among gait with multiple physical asymmetries. The results showed that mass at the distal end had a larger magnitude on overall gait asymmetry compared to leg length discrepancy. It also showed that the combined effects are varied based on the cancelation effect between gait parameters. The metric was also successful in delineating the differences of prosthetic gait and able-bodied gait at three different walking velocities.

## 1. Introduction

Human gait is a complex coordinated cyclic neuromuscular process that includes voluntary and involuntary aspects (Zijlstra et al., 1995). However, this cyclic process is frequently impaired following central nervous system damage, such as stroke, or physical changes, such as wearing a prosthetic. Physical and neurological changes often result in an asymmetric gait because the person's muscles and/or control actions becomes inherently asymmetric. Typically, human gait is represented by spatiotemporal, kinematic, and kinetic parameters obtained from analyzing motion capture and force plate data (Winter, 1995). The purpose of this study is to present a simple but versatile quantitative asymmetry metric that can be used to characterize the asymmetry of gait patterns as a whole.

Gait parameters offer quantitative data that can represent a person's gait. Using a quantitative data driven analysis offers an unbiased evaluation of the effects of multiple physical asymmetries that affect human gait. For this experimental study, the physical changes were selected based on the dynamic principles related to leg length and mass on a periodic system. Under appropriate conditions two dissimilar systems can be made to exhibit synchronized motion (Handzic et al., 2015). Handzic et al. demonstrated that two double pendulums with different masses at different locations and different lengths can exhibit symmetric motion. Human legs can be modeled as double pendulum systems, which allows for a simplified explanation of their synchronized dynamics (McGeer, 1990). Discrepancies in leg lengths and lower limb amputation disrupts natural propagation and dynamics that ultimately lead to asymmetric gait patterns. Further, asymmetric effects are also observed with changes in mass, such as the addition of external mass or a prosthesis. The study presented here also includes the effect of damping and stiffness at the knee to compare a larger range of physical changes that are not limited to altering the length and mass of limbs.

## 2. Background

Previous research about asymmetric physical changes reveal a range of different effects on a person's gait. The literature review for this study looked at various physical changes such as leg length discrepancy (LLD), the addition of mass at the distal end of the leg, amputation, and stroke. It is important to remember that although these physical changes affect every person differently, they can all be characterized using the asymmetries of biomechanical gait parameters. It is not uncommon to find similar effects on gait asymmetry with different physical changes. To illustrate these differences and similarities, this literature review also focused on prior quantitative gait metrics and the algorithms used to discern between different types of gait.

### 2.1. Gait patterns

Approximately 0.001% of people have some form of corrective gear due to LLD (Guichet et al., 1991). LLD may cause serious long-term consequences based on several variables such as the design of corrective devices, age, weight, posture, and level of activity (Gurney, 2002). An increase of 2 cm or 3.7% in leg length difference has dramatic overall gait asymmetry, especially in vertical reaction forces during push off and initial contact (Kaufman et al., 1996). Further, LLD causes abnormal changes in foot loading patterns and increases in joint torques/moments, which could lead to long-term effects (Perttunen et al., 2004). Finally, studies have also shown that LLD causes more overall strain on the body and leads to increased expenditure of energy (Gurney et al., 2001).

Limb mass, like limb length, plays an integral role in the dynamics of human walking. Adding mass on limbs, especially toward the distal end, brings about increases in metabolic activity and disrupts spatiotemporal symmetry (Browning et al., 2007). Adding mass at the distal end has been shown to force the user to change their walking posture by moving their arms in order to maintain balance (Donker et al., 2002). These effects may cause adverse changes in walking patterns in able-bodied symmetric individuals, but the addition of weight on the non-paretic limbs of stroke victims has shown improvement in walking speed, step length, cadence, and weight bearing in the paretic limb (Regnaux et al., 2008).

Studies show that prosthetic users exhibit less effcient and unnatural gait patterns (Gitter et al., 1995; Hoffman et al., 1997). This inefficiency is more evident in transfemoral amputees than transtibial amputees, which results in the users exerting a great deal of effort to compensate for unwanted motions (Huang et al., 1979). In some cases, simple solutions can correct irregular gait. When individuals with ataxia wore a 2 lb mass on their chest, unstable motions significantly decreased and the gait was more steady and efficient (Gibson-Horn, 2008). Since amputees are physically asymmetric, bringing about efficient and symmetric gait depends on multiple factors such as length, weight of prosthesis, type of socket, length of residual limb, etc. A study on unilateral transtibial prosthetic users shows that as the mass of the prosthetic gets closer to their intact shank weight, the subjects gait becomes more asymmetric (Mattes et al., 2000).

Stroke is one of the leading causes of disability among adults, affecting ambulation, performance of activities of daily living, communication, and cognition. Physical independence with respect to walking is characterized by improvement of walking function as defined by stroke survivors (Bohannon et al., 1991). However, only a minority of people (7–22%) are able to regain sufficient function to be considered independent community ambulators post stroke (Hill et al., 1997; Lord et al., 2004).

Gait retraining post-stroke typically focuses on two main outcome measures: velocity and symmetry. Walking velocity is used as an indicator of overall gait performance and can be used to differentiate the levels of disability among the stroke patient population (Perry et al., 1995; Lord et al., 2004). A gait speed of 0.8 m/s is considered the required minimum for community ambulation (Perry et al., 1995; Bowden et al., 2008), and typically people ambulate with a mean gait velocity of 1.14 m/s (Lord et al., 2004). Gait symmetry, in contrast, is used as a measure of gait quality (Dewar and Judge, 1990; Patterson et al., 2008). Normal gait measured among able-bodied individuals was found to be fairly symmetric in spatiotemporal, kinematic, and dynamic parameters with a range of up to 4–6% asymmetry between the limbs (Herzog et al., 1989; Titianova and Tarkka, 1995).

Gait after stroke becomes asymmetric (or hemiparetic) as a consequence of altered neuromuscular signals affecting leg motor areas, typically hyper extension at the knee and reduced flexion at the hip, knee, and ankle (Brandstater et al., 1983; Wall and Turnbull, 1986; Kelly-Hayes et al., 2003). Hemiparetic gait is characterized by a significant asymmetry in temporal (e.g., time spent in double-limb support) and spatial (e.g., step length) measures of interlimb coordination (Brandstater et al., 1983; Titianova and Tarkka, 1995; Balasubramanian et al., 2007). Propulsive force of the paretic limb is also reduced compared to the non-paretic limb, as are work and power of the paretic plantar flexors (Bowden et al., 2006; Balasubramanian et al., 2007). The significant decrease in propulsive force results in smaller overall step lengths, which in turn affects the patient's gait velocity. Finally, vertical ground reaction forces (GRFs) are decreased on the paretic limb relative to the non-paretic limb (Kim and Eng, 2003), reflecting diminished weight bearing and balancing capabilities by the paretic limb.

When an individual with an asymmetric impairment walks with symmetric step lengths, other aspects of gait become asymmetric, such as the forces in the joints (Carpes et al., 2010; Handzic et al., 2015), the amount of time spent on each leg (Kim and Eng, 2003), and other temporal variables (Sadeghi et al., 2000; Highsmith et al., 2010), all of which can be detrimental to efficiency and long-term viability. Understanding how symmetry affects function could change the fundamental nature of clinical gait rehabilitation. The results from this research could also help tailor rehabilitation treatments to target each person's specific impairment. An overall analysis of multiple gait parameters can bring equilibrium to the different, and sometimes conflicting, requirements of gait. In order to distinguish and characterize the effects of multiple gait parameters, we use metrics that consolidate and quantify the overall change in gait. This paper demonstrates the effectiveness of these quantitative gait metrics in classifying multiple physical asymmetric changes.

### 2.2. Gait metrics

Gait metrics have been in use clinically to evaluate a subject's progress throughout their rehabilitation process. These metrics can be classified based on the type of information required, which is of two types: qualitative (Steffen et al., 2002; McConvey and Bennett, 2005) and quantitative (Schutte et al., 2000; Schwartz and Rozumalski, 2008; Rozumalski and Schwartz, 2011). Most metrics focus either on kinetics or kinematics in order to categorize various walking patterns. However, there are some that can perform the analysis utilizing both kinetic and kinematic parameters (Chester et al., 2007; Hoerzer et al., 2015). Gait metrics have also employed statistical techniques such as principle component analysis (PCA) and singular value decomposition (SVD) to reduce the dimensionality of the biomechanical parameters (Muniz and Nadal, 2009). After processing the dataset, either the Euclidean or Mahalanobis distances (Muniz and Nadal, 2009) are found, which ultimately results in the score for the metric. Previous studies used Mahalanobis distances in conjunction with PCA to analyze kinematic and specific loading at knee joints. The precursor to this research study showed that the combined gait asymmetry metric (CGAM) used a symmetry index in conjunction with Mahalanobis distances. Without the restrictions of dimensionality reduction, CGAM served as a versatile gait asymmetry metric (Ramakrishnan et al., 2016).

## 3. Methods

In order to analyze multiple asymmetric physical changes using gait metrics, two distinct datasets were collected from eleven different types of physical alterations. The physical alterations include a prosthesis with two different sockets on an amputee, healthy individuals with eight combinations of leg length and ankle masses fitted to the non-dominant leg, and a stroke simulator. The distinct datasets for the alterations were collected on amputee and non-amputee populations. The amputee data was collected while walking at three different speeds on two types of sockets. The data collected on able-bodied subjects includes all of the perturbations.

### 3.1. Participants

The participants for this experiment consisted of 10 able-bodied individuals and a transfemoral prosthetic user who walked with two different sockets. Table 1 describes the subject population. Both studies were conducted under approved University of South Florida IRB protocols. The subjects provided both informed and written consents to take part in the experiments. The transfemoral amputee was selected because the subject was a high functioning transfemoral prosthetic user and can walk at speeds that are comparable to able-bodied subjects. For the data analysis, we consider the prosthetic user to be two different subjects because the change in sockets alters the subject's gait to a large extent. The study involved the subject walking at 3 different speeds using 2 different sockets: the vacuum assisted suspension (VAS) brimless socket (Klute et al., 2011) and ischial ramus containment (IRC) (Kahle, 2013).

**Table 1:** Participant information.

**Parameter**	**Able-bodied (10 subjects)**	**Prosthetic user (1 subject)**
Age (years)	Range: 18–28	36
	Mean: 22.2 and std: 3.2	
Height	Range: 155–196 cm	162.5 cm
	Mean: 171.2 cm and std: 11.44 cm	
Weight	Range: 48.08–82.55 kg	46 kg
	Mean: 69.2 kg and std: 11.34 kg	
Leg length	Range: 84–108 cm	84 cm
	Mean: 94 cm and std: 6.7 cm	
Walking speed	Range: 1.1–1.5 m/s	0.5–1.3 m/s
	Mean: 1.27 m/s and std: 0.13 m/s	
Gender	5 male and 5 female	1 female

The able-bodied individuals had no prior injuries that would alter their walking patterns. The subject's walking speeds were determined by a 10 m walk test after which the height, weight, leg length, and age of each individual participant were recorded. The participants were put through a series of randomized increments of leg lengths, addition of masses at distal end, and a combination of both effects on the same leg, which was the left leg in all cases (Muratagic et al., 2017). Table 2 shows the various perturbations of the experiment. Finally, the stroke simulator was fitted on their dominant side, which was the right leg for all participants.

**Table 2:** Experimental procedure.

**Trial type**	**Perturbation**	**Order**	**Side**
Prosthetic trial	0.5 m/s	In order	Right
	0.9 m/s		leg
	1.3 m/s		
Able-body	0.5 m/s	In order	N/A
	0.9 m/s		
	1.3 m/s		
Able-body	Leg length-non-weight-non		Left
	Leg length-big-weight-big		leg
	Leg length-small-weight-small		
	Leg length-small-weight-non		
	Leg length-big-weight-non	Randomized	
	Leg length-non-weight-small		
	Leg length-non-weight-big		
	Leg length-small-weight-big		
	Leg length-big-weight-small		
Able-body	With stroke simulator	In order	Right
	After stroke simulator		leg

### 3.2. Experimental apparatus

**Figure 1:**
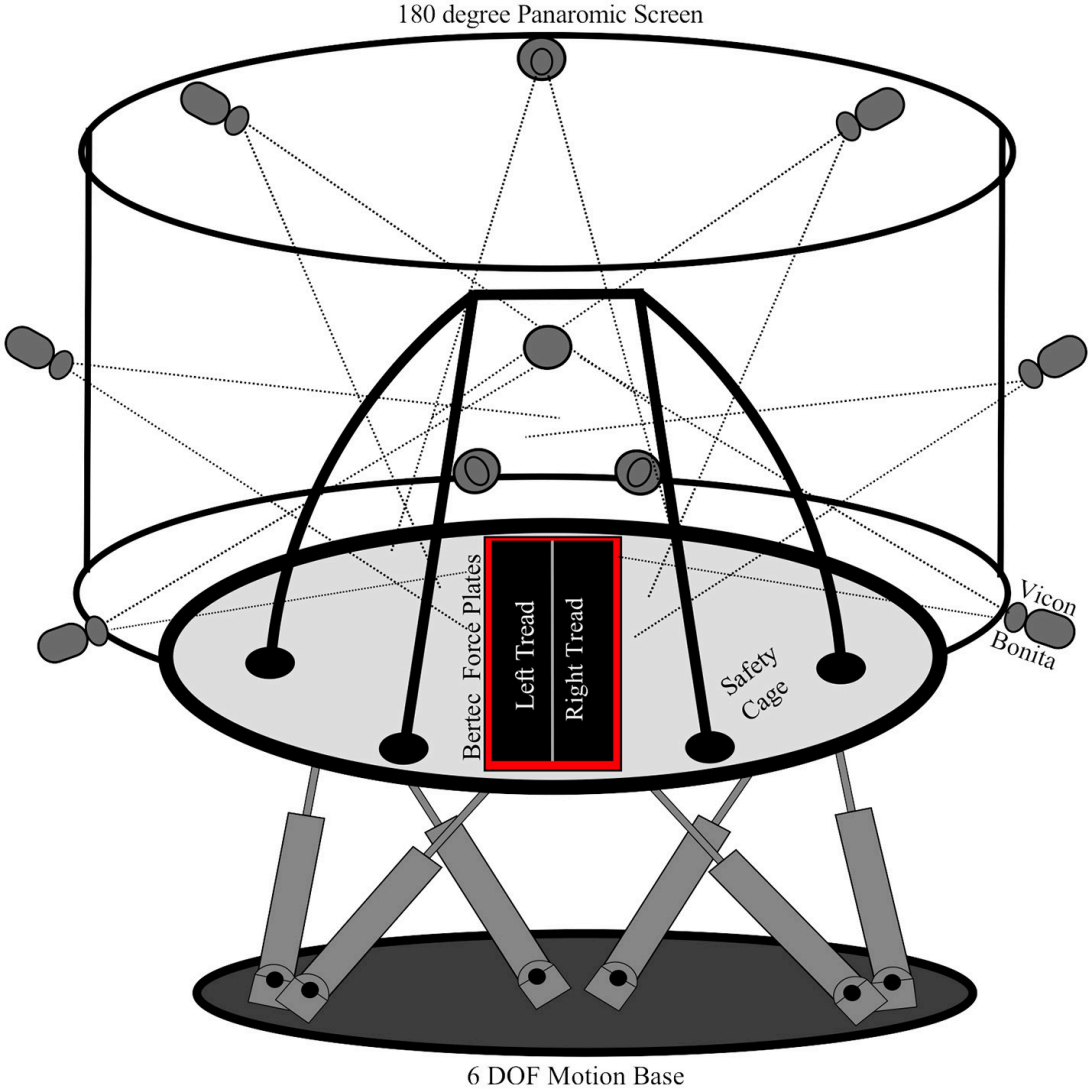
The computer assisted rehabilitation environment (CAREN) was used to collect the datasets for this research. The CAREN is equipped with a 10 Vicon Bonita infrared motion capture system, Bertec continuous force plates, split belt treadmill, 6 degree of freedom motion platform, and a fully enclosed safety cage. The subjects are secured to the safety cage of the CAREN for their safety.

The experimental data was collected in two separate trials, one on a single amputee and one on 10 able-bodied subjects. The motion capture and force plate data was collected using the Computer Assisted Rehabilitation Environment (CAREN), which was developed by Motek Medical, Netherlands, shown in Figure 1. The CAREN system incorporates a ten-camera Vicon (Edgewood, NY) motion capture system, 6° of freedom motion base developed by MOOG, immersive 180° panaromic screen for virtual reality environment, split belt treadmill, and continuous force plate systems developed by Bertec.

### 3.3. Experimental procedure

**Figure 2:**
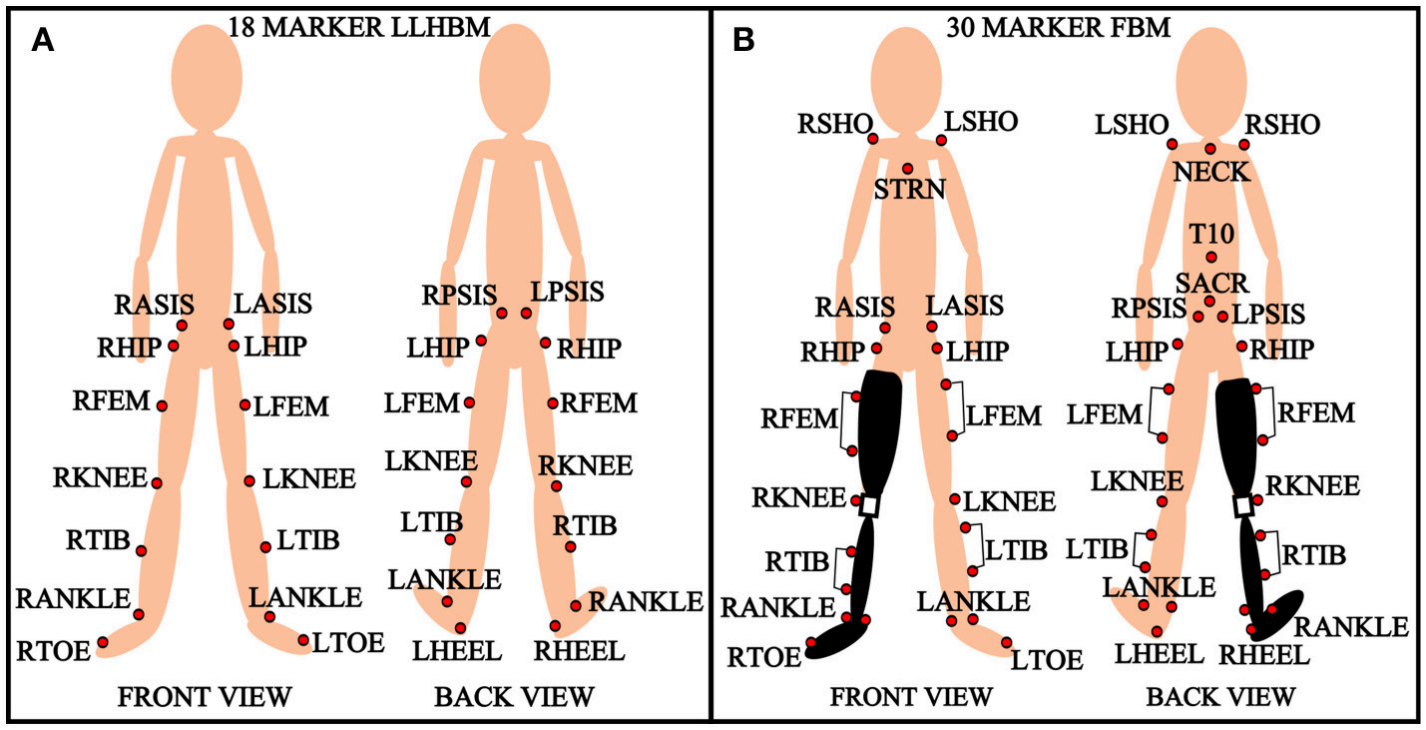
Marker setup. **(A)** 18 marker lower limb human body model (LLHBM) and **(B)** 30 marker full body model (FBM). R, right; L, left; ASIS, anterior superior iliac spine; PSIS, posterior superior iliac spine; FEM, femur; TIB, tibia; STRN, sternum; SACR, sacral wand marker; SHO, shoulder.

**Figure 3:**
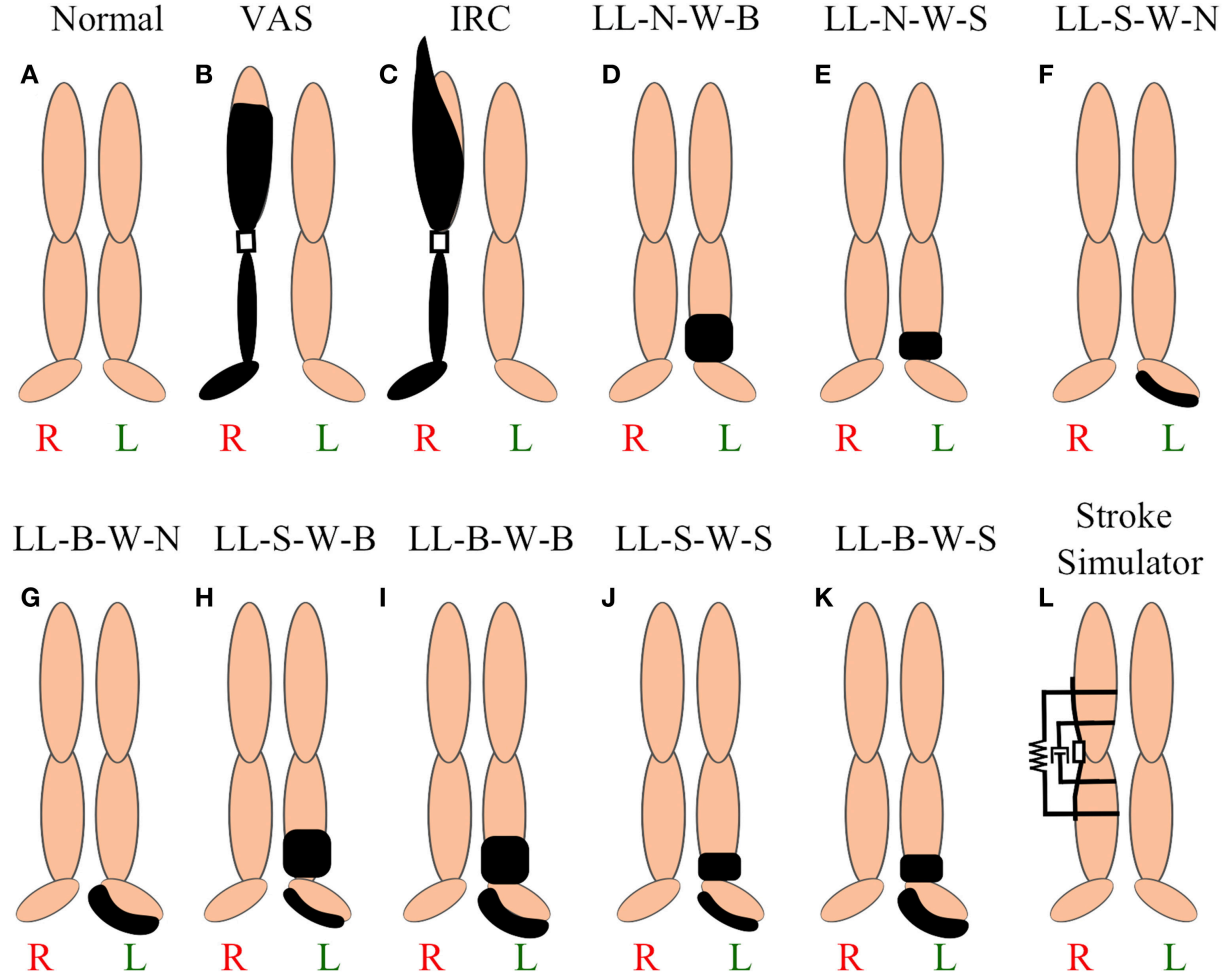
Various perturbations. **(A)** Able-bodied subject, **(B)** vacuum assisted suspension brimless socket, **(C)** ischial ramus containment, **(D)** no leg length and no weight (LL-N-W-B), **(E)** no leg length and small weight, **(F)** small leg length and no weight, **(G)** big leg length and small weight, **(H)** small leg length and big weight, **(I)** big leg length and big weight, **(J)** small leg length and small weight, **(K)** big leg length and small weight, and **(L)** stroke simulator (Lahiff et al., 2016) with rotational damper and torsional spring.

The data for the prosthetic trial was collected using 30 reflective markers, which can be seen in Figure 2. This marker set was used to collect extensive data on the lower and upper body dynamics of the amputee as part of another study. In this study we only use the lower limb markers out of the 30 for the gait analysis while the others are used in another analysis. The unilateral right transfemoral amputee used two prostheses, shown in Figures 3B,C, with different socket types, and all other components were identical. The amputee walked on both sockets at three different speeds: 0.5, 0.9, and 1.3 m/s. This was done to have a range of cadences that can represent both prosthetic and able-bodied users. The able-bodied subjects also walked at these three speeds for direct comparison. The socket systems used in the prosthesis were the IRC and VAS (Kahle et al., 2016). The IRC socket is designed to reduce pistoning and increase stability, but compromises on comfort while the VAS is designed more for comfort and aims to be dynamically efficient.

The able-bodied subjects were put through a series of 9 asymmetric changes, shown in Figures 3D–L, and a baseline symmetric gait, shown in Figure 3A. The subject's height, weight, leg length, and walking speed were recorded before beginning the experiment. The walking speed of the subject is recorded using a 10 m walk test over ground. This walking speed was the constant velocity at which the subject walked for the duration of the trials, except for the three different speeds discussed above. An 18 marker setup was used to capture the motion capture data for the able-bodied subjects. The marker setup for the lower limb is shown in Figure 2. The asymmetric physical changes are combinations of leg length changes and the addition of mass at the ankle. There were two levels of leg length alteration: a small height change of *L*_1_ = 27 mm and large increase of *L*_2_ = 52 mm. The small and large mass added at the distal end weighed *M*_1_ = 2.3 kg and *M*_2_ = 4.6 kg. The leg length was chosen to reflect a larger than 2 cm change in leg length which is detrimental according to literature. We used a linear relationship x and 2x to select the larger leg length. Similarly the mass was chosen based on a previous PDW study that used a linear selection method (Handzić and Reed, 2013). In addition to these changes, the subject's normal walking pattern was recorded before and after all the perturbations. The leg length and mass changes were added to the non-dominant leg of the subject to compound the asymmetric effect (Muratagic et al., 2017).

Following this trial the subject was fitted with a variable stiffness and damping knee orthotic device, which is also known as the stroke simulator (SS) (Lahiff et al., 2016). The SS is used to simulate the damping and resistance at the knee joint felt by stroke patients. The knee joint of the stroke patient has a damping effect due to the imbalance in control of the anterior and posterior femoral muscles. Stroke victims also experience stiffness/resistance to flexion of the knee joint due to the over excitation of the rectus femoris and lack of control of the posterior femoral muscles that render the knee in a constant state of extension. The device is a modified knee orthosis with a rotary damper of ζ = 8,898 g-cm-s/° for the damping effect and a torsional spring of *K* = 0.457 kg/mm for the stiffness effect. The device was fit on the subject's dominant leg. This is because the dominant limb is less coordinated and hence, exhibits the maximum asymmetric change (Sadeghi et al., 2000). The subject then walked with the SS for 10 min to adapt to the device's dynamics. Then the device was removed and the subject walks for another 2 min to measure any after effects due to the asymmetric change applied at the subject's knee.

### 3.4. Data analysis

**Figure 4:**
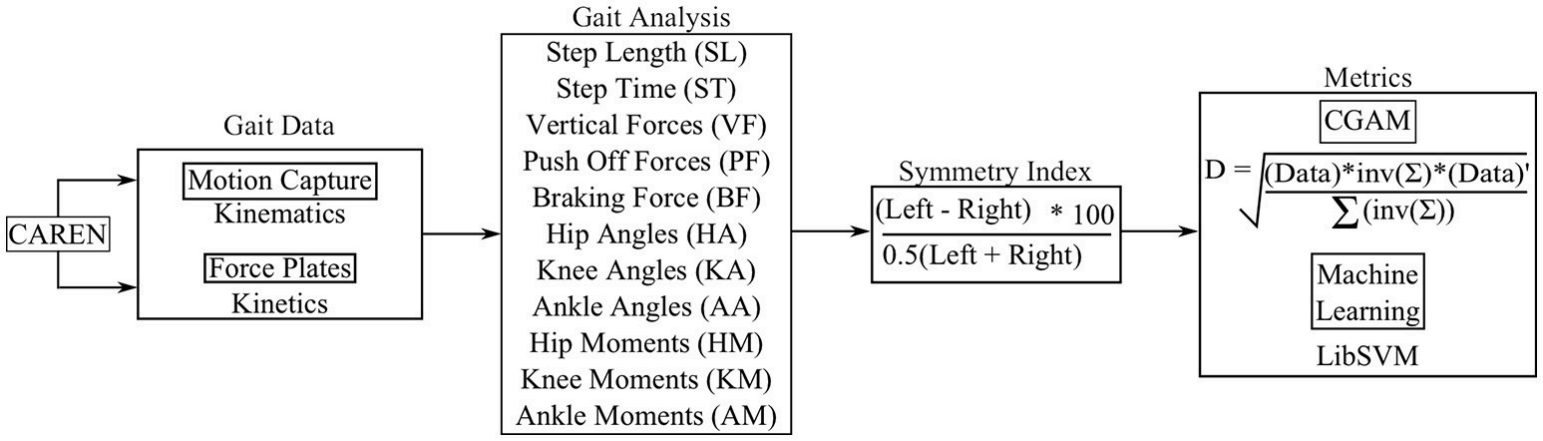
Procedure to acquire gait metrics.

The motion capture and force plate data gathered from the CAREN system is used to perform the gait analysis. The gait analysis was performed using a MATLAB script that evaluates the spatiotemporal, kinematic, and kinetic parameters from the raw coordinate and force data for each perturbation. Once the parameters are analyzed, their differences are evaluated for each step using the symmetric index formula (Herzog et al., 1989). This asymmetry data is then used to obtain the Combined Gait Asymmetry Metric (CGAM) (Ramakrishnan et al., 2016), which is a single number representing an Index/score for the level of asymmetry. The study further compares the CGAM to the machine learning grouping metric with the help of LibSVM library (Chang and Lin, 2011). Figure 4 shows the complete setup for the development of the metrics.

The CGAM is a simple metric that uses the Mahalanobis distance from ideal symmetry to the data points obtained from gait analysis. Mahalanobis distances are calculated in multi dimensional datasets such as the calculations performed on the 11 gait parameters, shown in Figure 4. The formula for calculating the CGAM distance is shown in Equation (1). The equation presented in this article is modified from the previous version of CGAM (Ramakrishnan et al., 2016). This formulation provides more of a weighted means approach to decrease the variability of results for the same gait parameters. This is achieved by dividing the original equation with the summation of the inverse covariance matrix. This method eliminates an extra step of dimensionality reduction that is carried out by algorithms such as Principle Component Analysis (PCA). Although PCA can help reduce the computational burden of multi dimensional datasets, it does so at the expense of losing information. CGAM's procedure analyzes the datasets without any loss in information and provides an overall perspective of the gait asymmetry based on biomechanical parameters. Further, the multiplication of the covariance matrix provides a weighted system that allows the metric to pick up on important changes in asymmetry among all the gait parameters.

(1) $$\begin{array}{r} CGAMDistance=\sqrt{\frac{\left(Data\right)*in\upsilon \left(\Sigma \right)*{\left(Data\right)}^{\prime }}{\sum \left(in\upsilon \left(\Sigma \right)\right)}} \end{array}$$

- CGAM Distance = Mahalanobis Distance from Ideal Symmetry
- *Data* = Matrix with n columns (11) and m rows (Number of Steps)
- Σ = Covariance of the Data.

## 4. Results

### 4.1. Calculating the CGAM score

**Figure 5:**
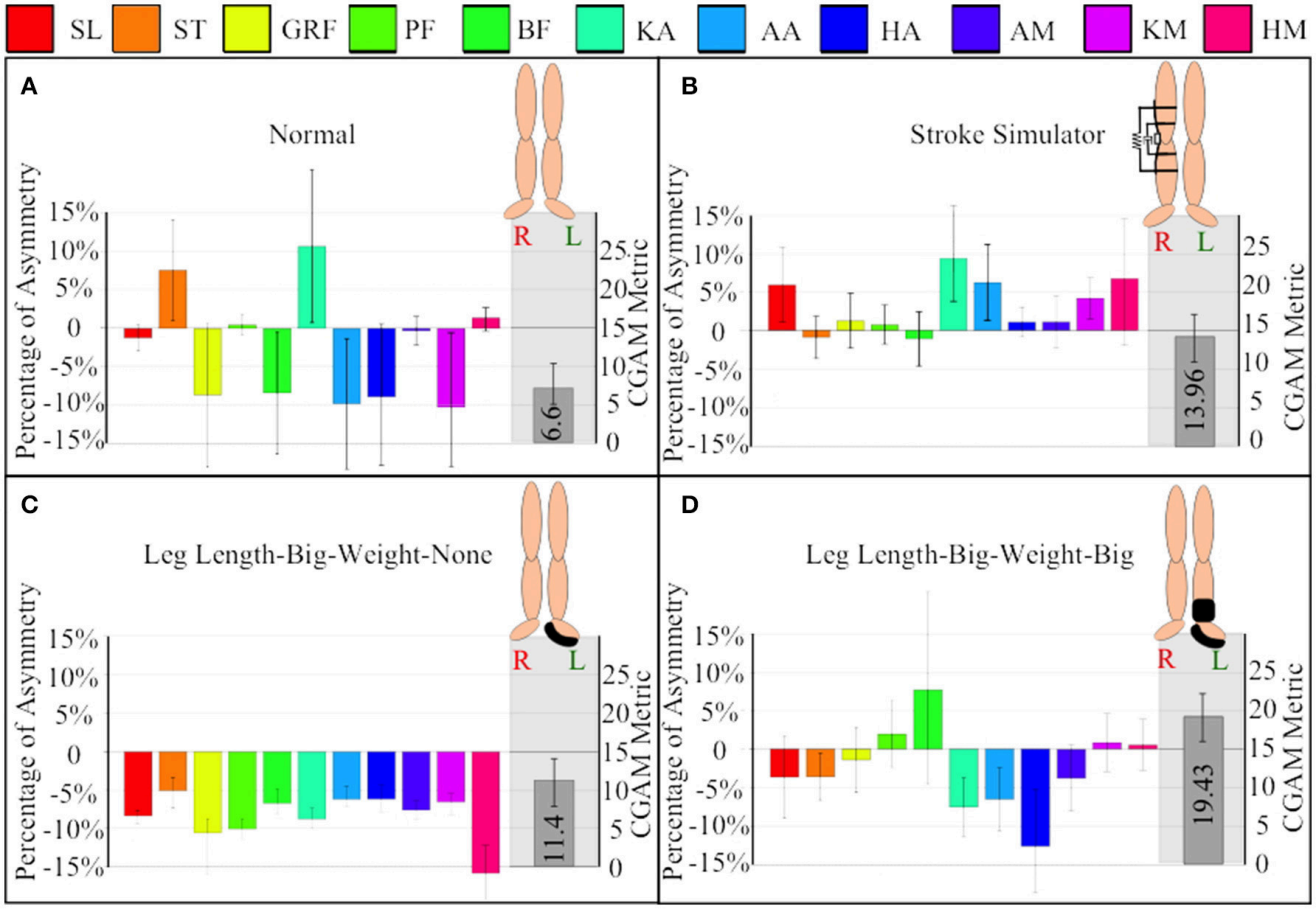
Comparing variation of mean, standard deviation, and CGAM metric among perturbations. **(A)** Normal walking without any alterations, **(B)** walking with SS or the variable stiffness and damping knee orthosis, **(C)** walking with big leg length and no weight addition at the ankles, and **(D)** walking with big leg length and weight. SL, step length; ST, step time; GRF, ground reaction forces; PF, push off forces; BF, braking forces; KA, knee angle; AA, ankle angle; HA, hip angle; AM, ankle moment; KM, knee moment, and HM, hip moment.

To further describe how the CGAM metric combines the gait parameters into one measure, the 11 gait parameters are shown in Figure 5 with their respective CGAM score for four of the gait alterations. An important aspect for interpreting this metric is the covariance of the asymmetry matrix, which serves to weight the measures based on how much variability is present. From Equation (1) it is clear that the covariance of the data plays a major role in calculating the Mahalanobis distances from ideal symmetry. The measures that have more variability get weighted less and more consistent measures are weighted more heavily. These weights generally account for the variations in magnitudes across all the parameters. For example, pushoff and braking forces tend to show much higher magnitude asymmetry than other measures, but they also show more variability; scaling them based on their variability makes the influence comparable to the other measures.

Even though the Stroke Simulator in Figure 5B looks to have low asymmetry on many measures, the variability is high on those measures. The high variability means that some steps have large asymmetry. Specifically the stroke simulator data shows a large increase in the step length and hip moment asymmetry that are part of the resultant increase in the CGAM score magnitude. In contrast a large hip moment asymmetry seen in Figure 5C, with large leg length increase on the left leg, does not increase the CGAM score as much since the other parameters are in the nominal range. It is important to keep in mind that the CGAM scores are measured from perfect symmetry, so even normal walking with no alteration has some asymmetry, as shown in Figure 5A. Figure 5D shows the combined overall effect of a large mass at the distal end and a large increase in leg length, which results in a larger score compared to large leg length only. Thus, the overall CGAM score is higher than the normal walking shown in Figure 5A, even though some of the normal walking averages are fairly asymmetric.

### 4.2. Comparison of alterations

**Figure 6:**
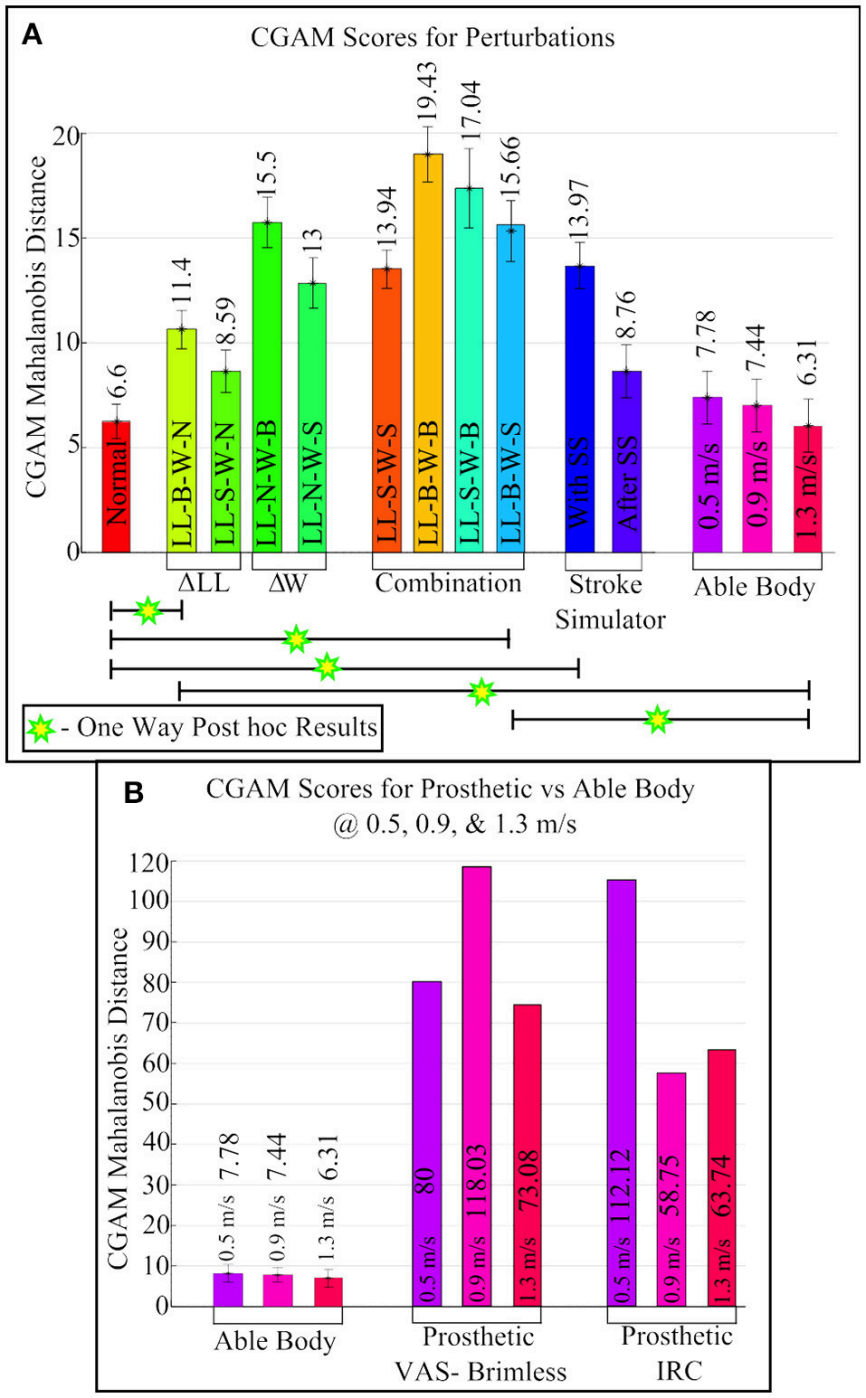
CGAM scores for all perturbations. **(A)** Able-bodied subjects with multiple physical asymmetries. **(B)** Comparison of Able-body subjects to prosthetic user at three different speeds (0.5, 0.9, and 1.3 m/s).

Figure 6A illustrates the CGAM scores with the alterations applied to able-bodied subjects, and Figure 6B illustrates the comparison of the scores between able-bodied individuals and the transfemoral prosthetic user walking with two different sockets. It can be seen in Figure 6A that the addition of mass at the distal end makes gait overall more asymmetric than an increase in leg length. The effect of combining mass and LLD showed that a large mass at the distal end and a small LLD had the overall highest asymmetry. This demonstrates that the largest physical asymmetry, which in this case was the large mass at the distal end combined with a large LLD, may not necessarily lead to the largest deviation in overall gait asymmetry. On closer inspection of the individual gait parameters, it was revealed that there may be a cancellation effect with the large change in leg length and hence the overall CGAM value was lower. When the subjects walked with just the larger leg length, the step lengths were more asymmetric than the step times; however, the step times were more asymmetric than step lengths when only wearing a large mass. This kind of behavior is illustrated with the different perturbations and hence, these opposite effects tend to cancel each other out which results in a lower CGAM value. A previous study conducted by Muratagic et al. (2017) found that there were no significant effects observed due to the combination of LLD and distal. However, the study also observed some cancellation effects due to the combination of LLD and mass which showed that there are potential combinations that could result in a balanced gait pattern.

The changes related to prosthetics also had significant effects, as shown in Figure 6B. Wearing the SS affected the gait of all able-bodied subjects and caused a similar level of asymmetry in this metric compared to an amputee wearing a prosthetic. However, speed affected the gait asymmetry, and there was one speed on each of the prostheses that the subject was not comfortable with. Another observation from Figure 6B is that the IRC socket is more consistent in overall gait asymmetry but the subject felt less pain using the VAS socket, and gait with the VAS has a better overall gait at a high velocity (Kahle et al., 2016).

### 4.3. Statistical analysis

A two-way repeated measures ANOVA analysis was performed with mass and leg length as independent variables and CGAM as the dependent variable. Mauchly's Test indicates that sphericity was not violated. The results of the ANOVA show that distal mass, *F*_(2, 18)_ = 19.15, *p* < 0.005, and leg length, *F*_(2, 18)_ = 5.72, *p* < 0.05, show statistically significance results in regards to CGAM. There was not a statistically significant interaction between the amount of mass added and amount of added leg length, *F*_(4, 36)_ = 0.20, *p* = 0.49. This is similar to the effects observed in our lab's previous study (Muratagic et al., 2017). Further, the *post-hoc* comparisons for mass revealed significant difference between no mass and both small and large mass conditions. There was a statistically significant difference between no length and the large leg length condition. This analysis method matches our previous study, and the conclusions are similar. However, this analysis excludes the stroke simulator and different speeds, so an additional one-way ANOVA was performed.

A one-way repeated measures ANOVA analysis was performed with all 14 of the gait patterns shown in Figure 6A used as independent variables and CGAM as the dependent variable. This analysis was performed to examine the individual differences of all the gait patterns, unlike the two-way ANOVA describe above that focused only on the added mass and height. Mauchly's Test indicates that sphericity was not violated. The results of the ANOVA show that there were a statistically significant differences in gait patterns, *F*_(13, 117)_ = 10.21, *p* < 0.0001. The *post-hoc* test results are shown in Figure 6A. The normal gait pattern is statistically significant to the perturbation with large leg length and small mass and the gait pattern with the stroke simulator. Similarly, gait pattern with the subject walking at 1.3 m/s showed statistical significant difference between perturbation with large mass and gait with stroke simulator. This was to be expected since 1.3 m/s is close to the average self selected speed of all the subjects.

### 4.4. Comparison to machine learning

**Figure 7:**
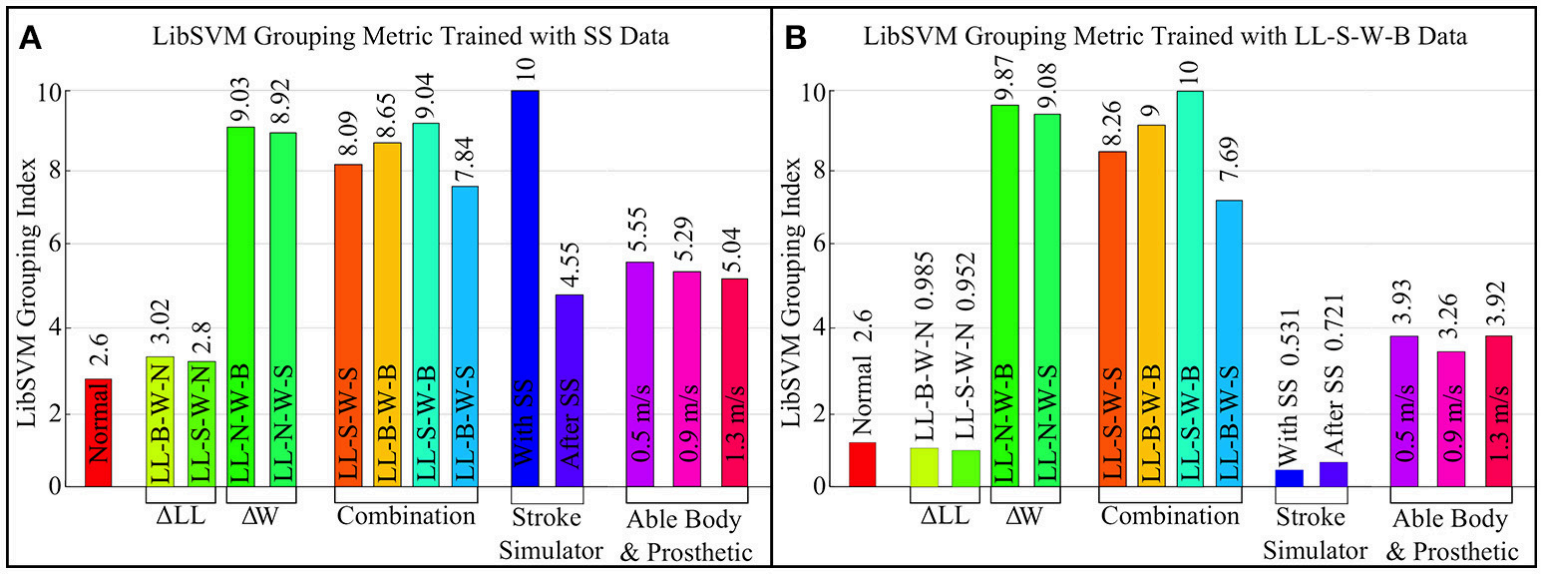
Machine learning (LibSVM) grouping metric using two different datasets for training. **(A)** Uses the asymmetry data for walking with stroke simulator. **(B)** Uses the LL-S-W-B data which was found to have the largest CGAM score notice the differences in grouping.

Machine learning has been used in data driven industries to find patterns in large amounts of disparate datasets. The two datasets that were collected during this study represent gait with multiple asymmetric changes and hence, can be used to find patterns. For this study the LibSVM library (Chang and Lin, 2011) was used because it is easy to implement and it is widely used for research data. The machine is trained using labels and a training dataset. The labels are long vectors with a single number and the training datasets are ground truths. In the case of this study the labels were 0 and 1. Label 0 was used for the perfect symmetry which is a zero matrix with 11 columns and multiple rows. Label 1 was used for training asymmetry data. Figure 7 shows the results of grouping predictions from 2 different asymmetry training datasets.

The pattern of the LibSVM grouping Index seen in Figure 7A is very similar to the pattern of the CGAM Mahalanobis distance in Figure 6A. Although the specific values cannot be compared directly because the modes by which they arrive at the results are inherently different, the trends highlight the differences between these two methods. The CGAM metric uses a simple Mahalanobis distance calculated from ideal symmetry while the more complex machine learning metric groups the data based on training datasets. LibSVM is not as reliable at this stage for being considered as a gait asymmetry metric because, based on the training datasets, the results vary substantially. This can be seen by comparing Figure 7A,B where the training datasets were different and the grouping predictions are completely different. This can be attributed to the different asymmetries present in the SS data and the weight/LL datasets. CGAM does not get affected by these differences and offers a more objective metric that can be used to classify the asymmetric changes. Another problem with Machine Learning as a metric is the requirement of large datasets.

## 5. Discussion

This study demonstrates a simple metric that can help classify physical changes in human gait using the asymmetries of gait parameters. The results discussed above show that the metric is able to successfully categorize the extent of asymmetric changes caused by different perturbations. For example the CGAM scores for walking with the SS, which is designed to cause asymmetric gait, has a significantly larger value compared to the value that was gathered for gait immediately after the device was taken off. The after-effects of the SS are also more asymmetric than a normal gait pattern, which shows that the individuals adapted to the SS. Classification of gait based on overall symmetry will help clinicians keep track of a subject's progress, such as pre- and post- physical therapy regiments. The SS can be examined as an impeding exoskeleton. Hence, the gait wearing the SS and after removing the SS are both asymmetric overall. Conversely, in robot-assisted locomotion therapy, the outcomes are expected to be more symmetric (Lo et al., 2010). CGAM could provide researchers the tools to measure the overall change in gait asymmetry and modify their rehabilitation techniques to induce better gait patterns. This approach is different from prior research practices that limited their study to either spatio-temporal, kinematic, or kinetic data.

Another approach is analyzing an individual's gait parameters separately. This method could reveal insights on specific comparisons, but the complexity increases with the number of gait parameters. It is difficult to determine if the gait has improved when separately examining 11 parameters. The CGAM could make this evaluation easier since it can be used to represent a range of gait parameters, and it is not just limited to the 11 parameters that were used in this study. The subsets of the gait parameters can be made to fit the requirements of the clinicians such as reporting on improvements in only spatiotemporal parameters or only in kinematics. For example, in a prior study with CGAM, only 5 gait parameters were used to analyze the data (Ramakrishnan et al., 2016). The parameters were step length, step time, vertical forces, push off forces, and braking forces. Using these 5 asymmetry parameters, the CGAM was able to classify the different perturbations of leg length and addition of masses on separate legs. Although this metric used 11 gait parameters, the two-way ANOVA showed similar results to the analysis performed using five gait parameters in the study by Muratagic et al. (2017). This leads to one of the avenues for future research which involves determining the minimum gait parameters required to represent a gait pattern. CGAM is designed to be used for any number of gait parameter asymmetries representing multiple forms of data. However, many research studies typically do not come equipped with a CAREN or similar system to gather large amounts of data. One of the advantages of CGAM is that it can be potentially used on limited availability of quantitative asymmetric data. We are exploring the boundaries of this metric to be able to benchmark it for standard protocols for gait analysis.

Consolidated metrics such as CGAM and Machine Learning offer a unique and simplified perspective into categorizing gait data between multiple asymmetric datasets. CGAM has the potential of serving as a benchmark in representing overall gait asymmetry using multiple different parameters. The multidimensionality that CGAM offers makes it versatile and as shown in this article we can assess multiple gait patterns with different causations. These metrics have to be field tested in clinical trials in order to be formally proposed for clinical use. It is important to remember that these metrics could direct researchers to help patients achieve a well rounded gait. A well rounded gait can be characterized as a sustainable gait that an individual adopts that has the least overall asymmetry, not just a decrease in one parameter. Some parameters would remain asymmetric so that other parameters could become closer to symmetry. In case of a person who is physically asymmetric, this would mean adopting a gait and posture that will have a balance between all the gait parameters. This adaptation of a well rounded gait will help a physically impaired person to sustain a long-term gait that may not necessarily be as symmetric as an able-bodied gait, but it is subjectively beneficial to their specific physical asymmetry. A well rounded gait will alleviate long-term problems caused by asymmetric forces and moments acting on the person's body.

In this study the 11 parameters were chosen because they represent important gait parameter information and have clear symmetry values between each limb. With both metrics it is clearly seen that the addition of mass at the distal end has a larger effect on the overall symmetry than leg length discrepancies. The combined effect of leg lengths and mass addition did not reveal a clear pattern but the results were as expected in most cases. For example, the combination of big leg length and mass had a slightly larger effect than small mass and leg length. However, the combination of a small leg length and big mass had a lot more deviation than big leg length and big mass. This is caused by the cancellation effects between gait parameters, which in turn resulted in a larger or smaller CGAM value.

There are some limitations associated with this method and study. There are many other gait patterns that were not discussed that arise from other gait impairments that should also be evaluated. Future studies can easily incorporate this metric in their analysis to compare the individual metrics to an overall picture of the gait. This will help in the generalization of this concept and also help to make the comparisons across different gait patterns more meaningful. This metric could also be optimized to find the most salient gait parameters to include; some of the ones used in this study may not be ideal and there may be others that are more beneficial to include.

The prosthetic gait at the three different speeds showed that the overall symmetry improves with increases in speed. It has been shown in literature that amputees achieve better spatio-temporal and kinematic symmetry at higher speeds, but at the expense of kinetic symmetry which can cause long-term degeneration effects (Nolan et al., 2003). We require a bigger patient population in order to gather all variations of prosthetic gait and leave that to future studies. The analysis provided in this study will improve further and can be more robust if a larger dataset from multiple patient population is used. The study presented in this article provides some proof into the efficacy of CGAM but it is limited by the small population size.

The CAREN is a versatile device that was used to collect all the data for this study and has been used in other similar studies (Ramakrishnan, 2014; Muratagic, 2015). To further understand the effects and dynamics of physical asymmetries, the split belt treadmill can be used to exaggerate asymmetries. Split belt treadmills are used to rehabilitate gait affected by hemiplegia by having the treads move at different velocities. This exaggeration of hemiplegic gait temporarily restores the person's gait closer to symmetry. However, successfully returning a person's gait to spatio-temporal symmetry does not necessarily guarantee an overall effective gait with a healthy ratio of symmetry between all gait parameters. To further explore how physical asymmetries combine, the split belt treadmill could be used in conjunction with an added mass and/or LLD.

## 6. Conclusion

Analyzing multiple physical asymmetries in one platform requires a special form of metric. This is because every perturbation of physical change that impairs an individual's gait has to be accounted for and kept track of following clinical procedures. The consolidated metrics such as CGAM and Machine learning can be quantitative data analysis tools that can help researchers keep track of a person's overall gait asymmetry. These metrics can be obtained using all gait asymmetry parameters such as spatio-temporal, kinematic, and kinetic or by using subsets and combinations of any or all of these parameters. This versatile platform allows researchers to have many options for generating metrics to represent the progress or regression of an individual over a period of training and time.

## Author contributions

TR designed and performed the experiments and analyzed the results. C-AL designed and tested the stroke simulator. C-AL also helped TR with the experiments and data analysis. KR advised C-AL and TR throughout the design, execution, and analysis. All authors participated in writing and revising the article.

### Conflict of interest statement

The authors declare that the research was conducted in the absence of any commercial or financial relationships that could be construed as a potential conflict of interest.
